# Commentary on the Hindu System of Medicine

**Published:** 1861-07

**Authors:** 


ART. IV.—*Commentary on the Hindu System of Medicine.* By T. A. WISE, M.D., Member of the Royal College of Surgeons, and of the Royal Medical and Chirurgical Society; Corresponding Member of the Zoological Society of London, and of the Philomathic Society of Paris; Bengal Medical Service. New Issue. London: Trübner & Co., 1860.

A WORK of this kind at first impresses us as a mere matter of curiosity, like some strange volume of travels; and the deepest emotion expected from its perusal is, perhaps, one of a scornful superiority, as seeing and feeling our vast advantages in enlightenment, in the inheritance which has devolved to us from a classic antiquity, and in the researches of modern science; and of an insulting pity toward the alleged half-civilized races whose records we are exploring.

Yet an account of Hindu medicine may be contemplated in other points of view. As a collateral light to the history of our science, a history, alas! too little studied in America, it is well worthy of the perusal of the practitioner who is not utterly broken down by labor, and incapacitated for extensive reading. Nor are such collections without their direct contributions to Europeo-American science: witness the Japanese discovery of acupuncturation. Without further parley, then, we bespeak for the volume before us great interest, arising from curiosity, from occasion to contemplate known facts in a new point of view, and from the hope of solid additions to our knowledge.

The American people, deeply penetrated as they are with the idea of perpetual progress, of the great superiority of this age, and of their own position, as being far in advance and setting an example, have nevertheless not shown themselves wanting in interest for the history of a high antiquity. The general and deep attention given by every class among us to the labors of Gliddon and Dr. Nott, as evinced by the great number of large classes, and the rapid sale of an expensive quarto, have surprised many; especially when they reflected that almost the only introduction to the subject, familiar to the great mass and acting as a source of interest, was the illustrations which these labors threw upon the Book of Genesis, and a few allusions and statements in Kings and the Prophets. To have presented to us a treasure full of the records and wisdom of "them of old time," drawn, most of it, from sources older than Moses, and discarding the more recent antiquity of Greece, to hold communion with the coevals of Zoroaster, and perhaps of Nimrod; to see sketched out a whole system of medicine from a departed world, and to hear the advice of men of the ages when God conversed with mortals face to face—to do this in regard to the manners and duties of a physician—such an opportunity might well offer grounds of independent attraction to Americans and to members of a learned profession.

Dr. Wise was in the service of the British East India Company, at Dacca, in the province of Bengal, British India; a large city which had been the capital, an honor now held by the new and immense Calcutta. Dacca lies between the Ganges and Brahmapootra, on a branch of the former, not far from the sea; in a *jungly* or forest marshy district, with much space having resemblance to our cane-brakes; and one which had long been under the concentrated influence of British power and proximity. These circumstances may have had some influence on his opinions.

His attention had been drawn, for many years, to the medical Shastras, or primeval religious medical works of India; which he found, in his neighborhood, to be in a state of slow decay from foreign influence, and which he much feared would soon be entirely lost. He made large translations of them from the extinct Sanscrit language, but soon found that he was grappling with a whole literature far beyond his leisure; and finally concluded to prepare and publish the present abstract.

According to his account, medical Sanscrit learning has fared much worse than the legal, mythological, philosophical, and historical productions of the same most ancient and wonderful school. Sir William Jones, amid all his fame, unequivocally denied the existence of any such science; (p. iv.,) and Mills, the historian, has imitated him. The only authors whom Dr. Wise cites as having shown their knowledge of its existence by writing on it, are as follows: Professor Wilson, in the Calcutta Transactions, and in the Oriental Magazine; Mr. A. Soma de Köros, of Hungary, in the Journal of the Calcutta Asiatic Society, in 1855; Dr. Hayne, in Tracts on India; Dr. Ainslie, in his copious work on the Indian Materia Medica; and Dr. Royle, in an essay on the Antiquity of Hindu Medicine, in 1838, and in able courses of lectures. We have not before us any of the productions of the continental press, although we know that such exist, learned, copious, judicious, and profound.

Yet it would appear that Mr. de Köros found extensive translations of the medical scriptures of the Hindus, into the language of Thibet, a country of which we hear so little. The Brahmins claim all the rank, confidence, and development to be derived from an antiquity of 3101 years before the Christian era; and the earliest collateral accounts ascribe to them the possession of all the estimation which they claim. Even the presumptuous Greeks, the Young America of that distant age, after the expedition of Alexander, treat the naked wise men, the gymnosophists of India, with deference and respect; and the treatises now extant, although become scarce, and, in Dr. Wise's part of India, extremely difficult of access, and generally preserved as conferring exclusive family privileges, are described as being most copious, dignified, and extensive. The Anglo-Saxons, like the other conquerors of India, and like most conquerors everywhere, have in general treated the fame, literature, and science of the vanquished with contempt and neglect. One of our townsmen, a few years ago, excited some laughter by expressing the hope, at a coming period, "to triumph over the archives of despotic Britain;" and the Hindu "archives" appear to have been the subjects of a pretty effective triumph. Not much wiser than their Tartar or Persian predecessors, and less wise than Alexander's Greeks, they have left a state of things which deceived Sir William Jones, and seems to have aimed at the oblivion of a whole mass of learning, of mighty departed empires, and of a whole age of this world. Later years have in part made up for this criminal negligence; but we are bound to acknowledge that but a portion of this retribution has come from the race of Odin. Splendid establishments have certainly been instituted by the East India Company; but excepting the writings of Sir William Jones, M. Silvestre de Sacy, and one or two other French, the great mass has been contributed by Germans. How hard that so little of this should have been done for medicine!

Our readers are, no doubt, too familiar with the character of what are called analytical reviews, when dealing with works of a copious and solid character, to expect anything approaching to a full abstract of their contents. Such a memoir is rarely possible within the space allowed, and when actually not impracticable, requires an expenditure of time and labor beyond what is ever allowed to journalists. We have looked over Dr. Wise's book, and will proceed to notice what strikes us as most likely to interest American physicians. As it will possess some convenience, perhaps we shall be allowed the privilege of using italic headings.

*Spelling and Pronunciation.*—The proper spelling of Sanscrit and other oriental words in English letters has formed a problem of much difficulty. The German mode, used much by comparative philologists, appears not to be in use among popular or general English writers; and the spellings in our use have generally been chosen by the various officers of the East India Company, who have frequently taken them by the ear, without any extended study. We have no other guide within our power, for the present article, than our author himself, whose directions we copy, as follows:—

"The consonants to be pronounced as in English.

"The vowels as in the Italian language. The long vowels will be distinguished by the following marks:—

| | | | | |
|---|---|---|---|---|
| *á* is to be sounded like | | | *a* in papa. | |
| *a* | " | " | " | *ay* in say. |
| *í* | " | " | " | *ee* in see. |
| *o* | " | " | " | *o* in so. |
| *u* | " | " | " | *oo* in too." |

We add, on the authority of oriental residents, that in *bh*, *kh*, and *gh*, the *h* must be separately pronounced after the preceding letter—these characters not expressing *v*, the Greek $\chi$, or soft $\gamma$. Thus, *Bh* conveys a sound somewhat resembling that used in the popular and comic expression, B'hoys; Khan, the familiar Tartar name for king, might be expressed K'han; and Ghaut, G'haut. For ourselves, we decline all opinion on these sounds.

The sacred Védas, as they first issued from the four mouths of Brahma, the awful Creator of all material things, were intended for instruction in the primitive age of the world, and contained all that was necessary for that period of innocence and purity. In the second age, one-third of the human race became depraved; and in the third, one-half of those who were then in existence. In the present degenerate period, the fourth age of Brahma's creation, and which has endured only 4960 years, besides other terrible marks of retribution from the gods for the crimes and impiety of our race, life is shortened to 100 years, and embittered with disease, which, in its various forms, was unknown to the primitive creation. It was then that Brahma, in pity to mankind, dictated to them additional Védas, for their instruction and comfort in their new and corrupted state. These are to be distinguished by the name of Upavédas; and among them, the Ayur-Véda contained all that was necessary for the preservation and restoration of health. Time, climate, and the crimes of men have done their work upon this heavenly gift, and all that we have left is composed of fragments preserved by commentators.

"Yet part no injuries of Heav'n could feel;
Like crystal, faithful to the graving steel—
Secure from flames, from envy's fiercer rage,
Destructive war, and all-involving age—
Their names, inscribed unnumbered ages past,
From Time's first birth, with Time itself shall last;
These, ever new, nor subject to decays,
Spread, and grow brighter with the length of days."

Eight treatises composed the first part of the divine original, and treated respectively of general surgery, of that of the face, head, and neck; of general diseases; of derangement of the intellect from the anger of the gods, or from demoniacal possession; of the diseases of children; of poisons; of the restoration of youth, beauty, and happiness; of alchemy; and of sexual weaknesses. The second Ayur-Véda treated of anatomy and dissection; of the causes of disease; of the preservation of health; and of a selection of remedies. The first physician, the individual to whom this revelation was vouchsafed, was Dakhsa, the Prajahpati, because he was an ocean of wisdom. His pupils, principally the "Ashwin" family, performed cures now considered miraculous; they healed the wounded of the battle between the gods and the giants, and replaced the fifth head of Brahma himself, after it had been cut off. Their success in curing paralysis induced the god Indra to study medicine.

After this, mankind became divided into sects; and precisely the same results ensued which are now visible from that cause in the United States of America. Men became ignorant, restless, unhappy, and afflicted with many painful and dangerous diseases in consequence. This is as should be expected; but what is odd, is, that a very modern-sounding step was adopted to relieve these evils. This was no other than a meeting of learned men of India, held by appointment in the Himalaya Mountains. The names of these medical philosophers, the "Rishis," are all given; but however deserving of glory they certainly were, we shall spare our readers the enumeration, notwithstanding our lesson in Sanscrit pronunciation. The names prove their extreme antiquity by having no Sanscrit etymology, and by their being replaced with a duplicate set, derived from the subsequently constituted Pantheon of the Hindus. We have not space, nor do we presume our readers have patience or curiosity, for the long names of the great physicians of this age of demigods.

Charaka, the historian of these occurrences, has, it is said, left a work "of the highest rank;" which certainly wants exact anatomical and pathological knowledge, and is often obscure. Simple remedies are described in it, with combinations of them, which do not reach the extravagant intricacy visible in those of some more modern volumes. His work, and that of Susruta, to be next mentioned, are actually the groundwork of the more recent medical systems; and these latter frequently display more imperfect descriptions than those of their celebrated masters, from ignorance of anatomy and etiology.

The next great name now frequently cited, is not exactly that of a demigod, but of a god, descended upon earth. When the ocean was churned by the gods, vatnas, or precious gifts to men, were produced; and among these was the divine Dhanwantari. This deity was instructed in the Ayur-Véda by the thousand-eyed Indra, "practiced medicine with great success in heaven, and became celebrated there." It seems unreasonable to us that the gods should suffer by disease, in consequence of the crimes of men. Dhanwantari descended upon earth, out of pity for mankind; became king of Benarès, and taught hygiene and medicine, by lectures, on earth; a rank, for a professor of medicine, somewhat higher than that of either Hippocrates or even King Thoth. Dhanwantari was petitioned by the association of Rishis to communicate his knowledge to Susruta; and the latter, under the dictation of his divine master, prepared an abridgment of the Ayur-Véda for human use; and this is the work to which we have already alluded as one of the two great classics of modern Hindu medicine.

The sacred Rishis were succeeded by other Munis, (sages,) and finally, under the institutes of Menu, the caste of Vaidyas was set apart for practitioners. Brahmins were permitted to study medicine for their instruction, Khetriyas for their own health, but nobody but a Vaidya was suffered to do it for his pecuniary support. The work of Charaka is considered the better for medicine, and that of Susruta for surgery and anatomy. The favorite compilation is that of Baboprukasa, who lived about three hundred years ago.

After all this mythological narrative, which is a mere sketch of Dr. Wise's more detailed history, we may try whether we can find anything like what mortals of these degenerate days call "common sense," amid the preceding instructions of the gods.

*Teachers of Medicine.*—Menu describes a very high character, indeed, for what a professor ought to be, both as regards learning, industry, morals, manners, and self-devotion. He must not be mercenary, but content with the honor and merit of bestowing knowledge. In many cases he receives no emolument, but may accept an allowance from the rich.

*Students.*—In all cases the student should be of a respectable and ancient family, and son of a practitioner, or of one who respects the medical profession. He should be inquisitive and observant; not covetous, zealous, or lazy; be a philanthropist, generous, and of an amiable and happy disposition. He should be active in his duties, and not fatigued by his studies; and possess gravity, a good memory, acute senses, and considerable acquirements. The teacher of such good students, after death, may expect to go to the heaven of Indra.

The progress of the student must be at first slow, commencing with the nomenclature, then acquiring single subjects, and lastly the whole system. The Shastras are not to be read on unlucky days; with numerous additions to the common list of such. After well completing his studies, and acting for himself, he must receive the permission of the Rajah to practice medicine.

*Physicians.*—The physician should possess a healthy body; he should keep his nails and beard short, his body pure, and clothes clean, and wear shoes and a small turban. He should carry an umbrella and a stick. He should possess a good memory, and be always amiable, cheerful, and collected. His language should be mild, candid, and encouraging, rather like that of a friend than of an acquaintance; and he should be always ready to assist the sick. He should possess a character for strict veracity, a calm temper, and the greatest sobriety and charity, and be a man of sense and benevolence. The patient should never be afraid of his physician.

Without the union of both theory and practice, his usefulness will be no better than the progress of a bird with one wing or a chariot with one wheel.

"Without a knowledge of books, he will be confused, like a soldier afraid in time of action, will be a great sinner, and should be capitally punished by the Rajah. On the other hand, a want of practical knowledge will impede his advancement; and his senses will be bewildered when he is called on to treat acute diseases. Such a physician will not be esteemed by the great, as he cannot practice with success when only instructed in half his duty."

"Some practitioners have many instruments and medicines which they do not know how to use. Such are calculated to deceive, and by their arrogant manners, and being without a knowledge of the Shastras, are enemies to mankind; and are called Chhadmachara. Those who possess the favorable qualities of physicians, without the necessary knowledge, are called Pratirápaka. The first two are sometimes allowed to practice by the neglect of the Rajah; and they may be known by their vanity and ill will toward the good physician. Such persons flatter the patient's friends, are diligent, take reduced fees, are hesitating and doubtful in performing difficult operations, and pretend that their bad success is caused by the bad attendants, etc. Such persons avoid the society of learned persons as they would a jungle." "Still some patients will be saved when under the care of such a physician."

There is much more of these "Medical Ethics;" some calculated to make us wish that Dhanwantari was more listened to than he would seem now to be in America. Thus of the

"*Messenger and Attendants.*—The person who is sent for the physician should not be of low caste, a fool, one of questionable veracity, or a great sinner. He should not appear before the physician distressed from fatigue, or by a rapid journey; or apparently fearful of the result of the sick person's ailment."

Of Dr. Wise's volume, fifty-three pages are devoted to anatomy and physiology, about thirty-two to descriptive anatomy, a few pages to the elements of which the body is composed, and the remainder is on therapeutic science, with its references to religion. One is fatigued with wonder in looking over such a volume, so strange to modern eyes appears this mixture of science, hypothesis, and mythology. The anatomical details appear wonderfully crude and wild; and yet they give evidence which cannot well be resisted that some who contributed to them had seen bodies opened, as well as had much surgical experience and some medical. The law which pronounces a Brahmin unclean if he touch a dead body, does not, according to Charaka and Susruta, render examinations of the corpse improper. A number of directions are given; and the body must be rendered putrid and scraped apart, instead of using the edge of the knife.

Brahmins can be purified after the unhallowed touch; and to a Vaidya, or one of the medical caste, if the rules of the sacred books are complied with, it is not unlawful.

A catalogue of vital parts, which it is peculiarly dangerous to wound, is given by Susruta; and here, with strange omissions, we still see the results of experience. Wounds near the great toe are apt to produce tetanus; those of the palm of the hand, hemorrhagy so severe as to require amputation of the arm. Depressed fragments of the skull are to be elevated or removed. Wounds of the upper part of the foot produce unsteadiness in walking; those of the ankle-joint, stiffness and lameness; in the middle of the thigh, fatal hemorrhage; just below the groin, great hemorrhage and paralysis of the limb; in the side of the os sacrum, paralysis; in the axilla, copious discharges of blood and pus, followed by death; in either side of the neck, death. The blood-vessels originate from the navel, as in the fœtus. Of the connective cellular membrane a description is given, which is almost accurate; of other parts, a mixture of striking truth with fanciful additions, which we will not occupy space in copying.

There are five elements: earth, water, air, fire, and ether. These constitute vegetable and animal bodies, by their combinations. The three principal humors are air, bile, and phlegm. From these are produced seven principal temperaments; three by the predominance of the three humors, three more by the influence of three pairs of them, and one by all three. Other and secondary temperaments, something like what the savages of these degenerate days have called idiosyncrasies, exist by the introduction of the qualities of various gods into the constitution of man; and another set, again, by those of animals, and even demons. An article on generation approximates very remarkably to the truth; and we have part of this on the authority of Siva himself. Very similar is the article on growth and the progress of life. If the ancient writings be not desultory, and deficient in strict arrangement, the fault must be with Dr. Wise; for his abridgment is certainly liable to that objection. Under Growth, we have a notice on Sleep, which we should have thought more appropriate to hygiene or physiology. Sleep during the day should always be avoided, and is a sin, except during excessive heat. "But children and old people, and those who have indulged in an excess of venery, who have consumption, drink much, or are much fatigued by traveling or other exercises, or those who are very hungry, or labor under indigestion, may sleep an hour (forty-eight minutes) in the daytime. If a person do not sleep during the night, he may take half the quantity of sleep during the day." Next follow accounts of the true nature of dreaming, drowsiness, yawning, languor, laziness, nausea, fainting, and swooning, and an article on digestion. Amid the detail of anatomy, there is a chapter on the excretions. After temperament, comes a chapter on ages and death. Natural death takes place at the end of one hundred years; and accidental death can be guarded against by care, medicine, and prayers. The pleasures of the senses mislead us to our death; therefore the wise should keep them under control. The holy man is the most indifferent to death, because he is the best prepared for it.

Death is only separation of the soul, and passage of the body to the five elements. Bodies are burned to prevent the defilement of these matters by interment in the earth. We should not regret the death of relatives and friends if they have passed through life with propriety: our grief is the offspring of our ignorance and selfishness. The transmigration of the soul is immediate; the liberated soul uniting itself at once with the seed of another being, and this of necessity.

"Beatitude is to be obtained by the coercion of the members, by abstaining from hurting or afflicting, or giving pain to sentient creatures; when the individual becomes fit for immortality." (Menu, 860.) "When the vital soul has been purified by the good deeds which have occurred in the body, it is absorbed into that supreme essence, the divine soul of all beings; which withdraws his energy; and placidly slumbers." "Let him not wish for death, let him not wish for life; let him expect his appointed time as a hired servant expects his wages. (Chap. vi., § 45, page 178.)"

About thirty-one pages are occupied with hygiene; and this part of the work is well worthy of the perusal of any man. The climate of our Louisiana resembles that of Bengal, in its combination of heat and dampness; and much of the surface of the United States partakes of the varied character of many parts of India. To this, it will be recollected, we are to add the actual living execution of the rules of ancient philosophers of whom we have read in books, perhaps most through the school of Pythagoras. We have, first, an account of the climates of the different parts of India, the hours of bathing and anointing, exercise and shampooing, clothing, the umbrella, the construction of houses, and diet. The articles of food are extremely diversified; and a large amount of animal nourishment is consumed. Sugar is strongly recommended as wholesome: a variety of oils are used, and largely for inunction.

*Water.*—"The Hindus were very careful about their drinking water, and ascribed the appearance of many diseases to bad water. They considered the water of wells, or natural springs in the sandy beds of rivers, as the most wholesome, as they promote digestion and strength. The river and fountain water at the bottom of high hills was considered less wholesome; and the most unhealthy was considered to be the water from brooks, and from tanks and reservoirs. This water was supposed to produce indigestions, obstructions, and lethargy, with a predisposition to fever."

Rain water, when clean, was considered beneficial to health.

Various *condiments* are used, many of them extremely hot.

*The grape* is considered cooling and aperient. Wine is made in the northern provinces; but in the southern, the heat forbids the necessary modification of the fermentation, the process being too rapid. In the Véda the use of wine and spirits is forbidden; but in the Tantra they are allowed, and the followers of Siva indulge in their use.

*Early hours* are universal, and the higher classes of Hindus are extremely strict in the performance of all their religious ceremonies.

To *Materia Medica*, forty-three pages of our author are devoted. This part of our subject is more known to physicians of the Western world. India has been investigated throughout by the ablest botanists; many of our most valuable drugs are brought from that region, and Dr. Ainslie's Materia Medica Indica has furnished a comprehensive account of them, with elaborate investigations. This review is not the place to pursue studies of the kind. Of plants familiar to us in the United States, we notice the following genera: pinus, betula, sida, bignonia, menispermum, ricinus, asparagus, asclepias, pothos, gossypium, hordeum, piper, hedysarum, anethum, datura, cannabis, andropogon, solanum, cupressus, vitex, cassia, saccharum, vallisneria, nymphea, panicum, ficus. All these are for diseases arising from excessive air, phlegm or bile. Emetics are obtained from the genera andropogon, sinapi, cassia, hedysarum, the melia azedarach, genera physalis, calamus, clitoria, anethum, plumbago, and acorus. Of purgatives, convolvulus, panicum, anthericum, asclepias, clitoria, plumbago, poa, saccharum, bignonia, ricinus, costus, cassia, euphorbia. Errhines are largely used; and include plants of the genera piper, nerium, hedysarum, achiranthes, iris, allium, betula, zinziber, citrus, saccharum, and ferula. Of diuretics we recognize only saccharum and pothos. External sialagogues are among others from the genera plumbago, sinapi, and zinziber. External stimulants, plumbago, asclepias, sinapi, and amomum. Among the uses of these last, is to increase the secretion of the male organs. Carminatives: piper, plumbago, rumex, ferula, and amomum. Astringents: lycopodium, panicum, menispermum. These are principally used for diarrhœa; and, for the healing of ulcers, and to stop hemorrhage, ficus, glycyrrhiza, diospyrus, and symplocus. We thought this catalogue would please our botanists and country practitioners.

Careful instructions are given for the choice of places in which to gather medicinal plants; and some of these rules seem dictated by a very sound botanical knowledge. Of the mineral productions, mercury is a good deal used; and very exact directions are given for the mode of preparation of it; which Dr. Wise has illustrated with several wood-cuts. A most serious prayer is offered up to Siva, before commencing the process; and we do not wonder at it. The ire of the "wrathful one" might well be supposed to interfere with it in some cases. The black sulphuret is the form most frequently used. The precipitatum album "cures rheumatism, and the eighty-three diseases of air, as well as the diseases of bile and phlegm." It is used so that sometimes the gums are affected. This preparation is also used for colic, fistula and other affections of the anus, diseases of the eyes, and general debility. It increases strength, appetite, and color—certainly not the Europeo-American experience. How much of this difference is owing to climate? Yellow sulphuret increases the appetite, and cures dropsy and dyspepsia. Cinnabar cures all diseases, even of the most fatal kind; removes weakness, improves the appetite and memory, diminishes the fat, and cures leprosy. Gold undergoes a great deal of preparation, and its different forms are considered as most valuable medicines, curing nearly all diseases, even those in which other remedies have been used without any good results. They increase memory and restore the vigor of manhood. How natural and consonant to the temper of man is the stimulating treatment! An oxide of gold mixed with lead, sulphur, and quicksilver is considered by some practitioners as an excellent tonic, improving vision, reducing the bulk of the body, good for consumption, for pregnant women and for children, and useful in diseases of air, bile, and phlegm.

Copper is useful in fevers, particularly intermittent, diarrhœa, spleen, and diseases of the liver and blood, in leprosy, colic, piles, and indigestion, and for strengthening the teeth. Lead is recommended for leprosy, ulcers, gonorrhœa, and chronic diarrhœa. Dr. Wise says nothing of colica pictonum and palsy. Tin diminishes fat, and the diseases of phlegm; is an anthelmintic, and cures gonorrhœa and jaundice. It is to be avoided in diseases of air. Zinc resembles copper in its effects. Antimony is applied as a collyrium, and to the edges of the lids, to increase the brilliancy of the organ. It is also used as an emetic in the beginning of fever, and enters into some compounds. Iron, in the sulphate, is of use in gonorrhœa, diseases of the urine, worms, and various diseases of the bile and phlegm, and, in combination, for improving the strength. An oxide, which makes us think of the black oxide, is an excellent tonic, improves digestion, and removes all diseases. There is no alterative so good as this. Yellow sulphuret of arsenic is very celebrated in the black leprosy and in fever, and improves the color of the body. Red arsenic is used for diseases of phlegm, for asthma, etc., and is tonic. White oxide is used, in the dose of the fourteenth part of a grain, in conjunction with aromatics, to check obstinate intermitting fevers, and in glandular and leprous affections.

We run hastily over the employment of gems and corals. Of the first the dose must be small, and, we should think, the effects imaginary. Of animal bodies the classification is taken from the different parts and fluids of the same body, and has not interested us. The most active remedies from the animal kingdom, we should think, were neglected. Thus, we did not observe musk or cantharides; although the confused character of the chapter, which we fear must be charged upon the favorite of the gods, Susruta, *ϑεõις 'επιεικέλῳ*, may have caused us to overlook them. They cannot have had the repute they enjoy among us barbarians of later ages.

The remainder of Dr. Wise's volume is occupied, for 82 pages, with pharmacy; for 36, with surgery; for 219, with the diagnosis and treatment of internal diseases; for 15, with midwifery; and for 2 with diseases of children. It would give us great pleasure to go over the whole of this, as far as we could analyze it, or even to make copious abstracts of parts of it; but this our space forbids, and we hope that the matter with which we have filled our preceding leaves has not been uninteresting. It has at least deeply interested us.

The division of diseases into sthenic and asthenic, according to Dr. Wise, "appears to have been an early opinion among the Hindus," and "is now generally believed over all the Asiatic nations." He does not quote Charaka for it. On the other hand, this favorite of the gods, who preceded Susruta, the surgeon, and even the god or Deva, Dhanwantari, and obtained his wisdom from the mighty Indra, the great authority for internal pathology, appears to have been a humoralist. He seems to have referred the production of many diseases, and the modification of all, to the relative predominancy of the three principles, air, bile, and phlegm. The agency of air corresponds most nearly with what we have been accustomed to consider a nervous temperament, combined, however, with some cases in which distention by air is obvious to the senses. The bilious and phlegmatic derangements do not differ very widely from what we have been accustomed to ascribe to the temperaments which bear the corresponding names; including the actual production of bile and mucus in quantities and circumstances beyond their usual and healthy custom.

As far as we have been able to examine the volume, the whole of this mass of science seems to bear consistently the same character—the results of undeniable and extensive observation, with a constant effort to make them agree with preconceived ideas derived from a different source. In fact, in considering the best parts of this body of doctrine, we are constantly led back to the idea of its origin in a still remoter antiquity, and of its elements and practice being remains from a destroyed empire, in which such knowledge was derived from investigation and experience, and had body and system before any dream was entertained of consigning it to posterity under the sanction of a ceremonial religion. The very fact, mentioned in the earlier part of this review, that the names of the learned men who held the meeting in the Himalaya, as originally given by Charaka, have no Sanscrit or other known etymologies, and cannot be explained, but were subsequently replaced by another set of appellatives, for which derivations can be found by means of dictionaries and catalogues of the Hindu pantheon, as with the giant in Homer,

"Whom Gods Briareus, men Ægeon, name,"

—this fact seems to point to some forgotten period of the world, whose prosperity and history were alike annihilated, but which gave time and opportunity for the development of the Sanscrit language to that wonderful perfection ascribed to it by scholars, and for the production of the rational and useful part of this system of medical science. There is room in history for such occurrences. Genesis, as well as Homer, is full of such reduplications of names. The history may have been contemporary with the earliest dynasties of the old empire of Egypt, with the sovereignties of the nine kings who warred in the Vale of Siddim, with Ur of the Chaldees, when Abram and Lot emigrated from it, and with the reign of the gods in China. It is easy to imagine how wise men, moved by commiseration for mankind, might commit it to posterity under the sanction of religion, and ascribe to it the blessings, authority, and parentage of the gods in whom their people confided. These might be the effectual means to preserve it through the shock of warring empires, the devastation of continents, and the ruin of civilization. In this mode might the rational and humane wisdom of an earlier age descend to form what seems to us such an astonishing and incomprehensible mixture with childish fables, long-continued practical error, and unwillingness to receive improvement at the hands of others.

"The thing that hath been, it is that which shall be, and that which is done is that which shall be done; and there is no new thing under the sun. Is there anything whereof it may be said, 'See, this is new?' it hath been already of old time which was before us."

